# Does therapy always need touch? A cross-sectional study among Switzerland-based occupational therapists and midwives regarding their experience with health care at a distance during the COVID-19 pandemic in spring 2020

**DOI:** 10.1186/s12913-021-06527-9

**Published:** 2021-06-15

**Authors:** Verena Klamroth-Marganska, Michael Gemperle, Thomas Ballmer, Susanne Grylka-Baeschlin, Jessica Pehlke-Milde, Brigitte E. Gantschnig

**Affiliations:** 1 Institute of Occupational Therapy, School of Health Professions, ZHAW Zurich University of Applied Sciences, Winterthur, Switzerland; 2 Institute of Midwifery, School of Health Professions, ZHAW Zurich University of Applied Sciences, Winterthur, Switzerland; 3 Department of Rheumatology, Immunology, and Allergology, University Hospital (Inselspital) and University Bern, Bern, Switzerland

**Keywords:** Tele-health, Tele-care, Tele-rehabilitation, Tele-monitoring health professions, Videotelephony, Technology acceptance, Occupational therapy, Midwifery

## Abstract

**Background:**

The COVID-19 pandemic impedes therapy and care activities. Tele-health, i.e., the provision of health care at a distance (HCD), is a promising way to fill the supply gap. However, facilitators and barriers influence the use and experience of HCD for occupational therapists (OTs) and midwives.

We identified use of services and appraisal of experiences of Switzerland-based OTs and midwives regarding the provision of HCD during the lockdown as it pertains to the COVID-19 pandemic in spring 2020. 1. Hypothesis: Profession, age in years, and area of work have a significant and meaningful influence over whether HCD is provided. 2. Hypothesis: Profession, age in years, area of work, possibility of reimbursement by health insurance, and application used have a significant and meaningful influence on the experience of HCD.

**Methods:**

In a cross-sectional survey, 5755 OTs and midwives were contacted to fill out an online questionnaire with 13 questions regarding demographic information, use of HCD, and experiences while providing the service. Eleven potential facilitators and barriers and areas where there was desire for support were identified.

**Results:**

The questionnaire was completed by 1269 health professionals (response rate 22.5%). 73.4% of responding OTs (*n* = 431) and midwives (*n* = 501) provided HCD during the COVID-19 pandemic lockdown. Profession and area of work had a significant influence on whether HCD was provided. Age only had a significant influence on the use of videotelephony, SMS, and chat services.

OTs experienced HCD significantly more positively than midwives (log odds = 1.3; *p* ≤ .01). Video-telephony (log odds = 1.1; *p* ≤ .01) and use of phone (log odds = 0.8; *p* = .01) were positive predictors for positive experience, while use of SMS (log odds = − 0.33; *p* = .02) was a negative predictor.

Among OTs, 67.5% experienced HCD as positive or mostly positive, while 27.0% experienced it as negative or mostly negative. Among midwives, 39.5% experienced it as positive or mostly positive, while 57.5% experienced it as negative or mostly negative. Most respondents desired support concerning reimbursement by health insurance (70.8%), followed by law and data protection (60.4%).

**Conclusions:**

HCD during the early COVID-19 pandemic was generally perceived as positive by OTs and midwives. There is need for training opportunities in connection with HCD during the COVID-19 pandemic.

**Supplementary Information:**

The online version contains supplementary material available at 10.1186/s12913-021-06527-9.

## Background

Coronavirus disease 2019 (COVID-19) is caused by severe acute respiratory syndrome coronavirus 2 (SARS-CoV-2) infection. As SARS-CoV-2 is highly contagious, the World Health Organization (WHO) announced the spread of COVID-19 as a pandemic in March 2020. Most states worldwide decided in March 2020 to substantially lock down public life to prevent the spread of the disease (date of lockdown in Switzerland as communicated on March 13, 2020 began on March 16, 2020). Those who tested positive for COVID-19 and vulnerable persons (e.g., > 65 years of age) were isolated. Unfortunately, those who require rehabilitation are often made vulnerable by the disease (e.g., 90% of stroke clients in Switzerland are 65 and above) [1]. Making matters worse, the pandemic resulted in very early inpatient discharge and a suspension of rehabilitation services in outpatient settings which decreased the access to rehabilitation services and their availability [2, 3]. As a result, occupational therapists were faced with providing interventions for an increasing number of clients isolated for their own protection or the protection of others.

The COVID-19 pandemic caused additional strain through a high prevalence of disease, limited resources, and staff under pressure [4].

This being the case, birth dates could only be rescheduled to a limited extent. At the beginning of the pandemic, studies provided contradictory evidence whether pregnant and postpartum women were at increased risk during the infectious disease outbreak [5, 6]. The increased risk of infection for postpartum women in hospitals and limited possibilities to receive visitors led to early discharge, consequently increasing the need for outpatient care. However, to avoid spreading the disease, midwives were challenged with quickly reducing in-person antenatal, intrapartum, and postpartum midwifery care, replacing it with remote care.

Tele-health, i.e., the provision of health care at a distance (HCD), might be suitable for continuing therapy under these circumstances [7]. The use of HCD has been described in a number of areas of work, including the outpatient area, the inpatient sector, the home environment, and school settings [8]. HCD reduces physical contact between vulnerable persons and the environment while enabling interaction with persons in strict isolation, therefore facilitating intense and long-term treatment [9]. Communication technologies typically used include phones, short message services (SMS), videotelephony systems, and application software programs (apps). HCD allows for remote evaluation, assessment, monitoring, prevention, intervention, supervision, education, counselling, and coaching [8]. In rehabilitation, there is evidence that remote interventions (“telerehabilitation”) have either better or equal salutary effects on motor, higher cortical, and mood disorders as well as quality of life compared with conventional in-person interventions [10–12]. In midwifery, tele-care, mainly in the form of videotelephony, had already been successfully applied for different purposes. These included education (e.g., for childbirth and parenting antenatal), assessment (e.g., in early labor), clinical supervision (e.g., diabetes control), peer support, and case review [13–16].

Although HCD may represent the first option for the treatment of people in isolation, most occupational therapists (OTs) and midwives were not adequately prepared to change therapy settings and care from in-person to remote during the rapid lockdown caused by the COVID-19 pandemic. Measures associated with the successful implementation of tele-health services such as education and training, as well as administrative and technical support could not be implemented in time. Little scientific literature exists to support health providers with information about the successful application of tele-health. To our knowledge, only Australia’s Nursing and Midwifery Federation has developed HCD professional practice standards and guidelines written specifically for nurses and midwives which provide recommendations regarding communication, technology, consent, privacy, and confidentiality [17].

In the literature, facilitators and barriers for successful implementation of HCD have been described [18, 19]. These include infrastructure, knowledge about apps, law and data protection, reimbursement by health insurance, federal and cantonal ordinances, client needs, client requirements, suitable methods and their effectiveness, communication methods, and the examination and treatment processes.

To integrate HCD technology into routine practice during the COVID-19 pandemic and in the case of future pandemics, it is necessary to understand by whom, how, and in which areas of work HCD is provided, and to evaluate the experiences and perceptions of health care professionals regarding the appropriateness and meaningfulness of HCD. We were therefore interested in the experiences and attitudes of health professionals who normally have physical contact with their clients and regularly care for them in 1:1 settings. OT and midwives in Switzerland were interested in exploring the experience of HCD during the COVID-19 pandemic. Their work before the pandemic has been predominantly performed in physical presence. Moreover, touch is an essential part of the work of OT and midwives, whether in guiding clients within therapies (indispensable for OT in particular) or in physical check-ups (midwives). Until before the pandemic, therefore, both professions offered only very limited HCD. The aim of the survey was to identify the use of services and to appraise the experiences of Switzerland-based OTs and midwives regarding the provision of HCD during the lockdown due to the COVID-19 pandemic in spring 2020. The study presented helps to understand facilitators and barriers for successful implementation of tele-health applications in occupational therapy and midwifery and facilitates the development of workshops and guidelines to support health care providers during COVID-19 pandemic [14].

The two professions differ in the type of services (e.g., tele-therapy, tele-counseling, tele-monitoring) they provide. Also, HCD may be seen as more appropriate for services typically provided in certain areas of work, e.g., in an outpatient setting or at clients’ homes. Furthermore, it is reasonable to assume that health professionals are more likely to provide services they can be reimbursed for [18]. It’s also worth mentioning that younger professionals may be more competent in using a wider range of communication technologies [20].

## Methods

### Study aim

We hypothesize that profession (i.e., OT or midwife), age in years, and area of work have significant and meaningful influence on the provision of HCD.

Furthermore, we hypothesize that profession, age in years, area of work, possibility of reimbursement by health insurance, and application used (i.e., phone, email, chat, SMS, videotelephony apps, and services) have a significant and meaningful influence on experience of a given HCD service by health care providers.

### Study design

In our reporting of the survey we follow the CHERRIES guidelines that were mainly designed for web-based surveys [21, 22].

### Study setting

The cross-sectional survey was accessible from May 11, 2020 to May 26, 2020. The population of interest encompasses OTs (registered members of the Swiss Professional Association of Occupational Therapists (“ErgotherapeutInnen-Verband Schweiz”, EVS, *N* = 2454 members) and midwifes (registered members of the Swiss Federation of Midwives “Schweizerischer Hebammenverband”, SHV, *N* = 3301 members) professionally registered in Switzerland. We collected data by means of an online survey. Representatives of the respective professional associations contacted OTs and midwives directly by email and provided them with information about the survey as well as a link to the online questionnaire. Participants received no financial incentives.

We used the survey creation platform www.unipark.com to compile an online questionnaire [23]. The platform allows for the creation and testing of surveys and provides an online link where it can be accessed. A data center situated in Germany hosts the platform which is certified by the German Federal Cyber Security Authority BSI and compliant with the ISO 27001 data safety and protection regulations [24]. Multiple participants were able to fill out the survey using the same IP-address. This was deemed necessary as the survey targeted health professionals who might share an IP-address or even an office computer in their places of work. To fill out the survey multiple times in the same browser, cookies had to be erased first. After the running time of the online survey had ended, we exported the e-data and imported it into SPSS.

#### Questionnaire

Before filling in the questionnaire, we informed potential participants about the purpose of the study, which stated:

*With the COVID-19 pandemic, the digital future has become the present. In the short term, examinations, treatments, and therapy with physical presence have been replaced by treatments at a distance. In doing so, health care professionals (i.e., OTs, midwives) must not only treat clients effectively, but must also observe the legal requirements. We would like to ask you a few questions about your experiences during the COVID-19 pandemic. Based on your answers, we would like to develop recommendations for you in cooperation with the professional associations.*

Potential participants were provided with information about the approximate length of time to fill in the survey (i.e., 10 min). The function and contact information of the two main investigators were provided.

The questionnaire consisted of 13 questions. The demographic section asked for information about the following: age in years, work experience in years, profession (i.e., OT or midwife), the field of activity of the institution/organization of employment, specific outpatient area (e.g., practice) inpatient sector (e.g., hospital, birth center, retirement home), the home environment, or school setting. Multiple answers were possible here.

If a participant confirmed that during the COVID-19 pandemic, he/she performed HCD (i.e., necessary urgent examinations, treatments, and therapies at a distance) rather than in his/her office or the client’s home, the following questions concerning which media were used (phone, email, chat, SMS, videotelephony apps and services) as well as their respective suitability for examinations, treatments, and therapies at a distance (rated on a Likert scale from 1 = negative, 2 = rather negative, 3 = rather positive, 4 = positive, 5 = I do not know). If he/she did not confirm this, the questionnaire skipped ahead to a question about perceived advantages and disadvantages of HCD. If participants confirmed that they had used videotelephony, they were then asked about which specific videotelephony apps and services they used when providing HCD. Possible answers were *Doxy.me, Skype, Viber, WhatsApp, MS Teams, FaceTime, Messenger, Zoom* or others. *Doxy.me* is a US-based videotelephony and instant messaging service geared especially towards telemedicine. *Skype* is a videotelephony service that also allows audio calls and instant messaging. *MS Teams* is a workplace-focused collaboration platform that includes instant messaging, video- and audio telephony, and file sharing services. Both *Skype* and *MS Teams* are owned and operated by Microsoft Inc. *FaceTime* is a VoIP (voice over internet protocol) and videotelephony service operated by Apple Inc. that exclusively runs on macOS and iOS devices. *Whats-App* and *Viber* are VoIP and instant messaging software applications that allow for video calls. *WhatsApp* is owned by Facebook Inc., while *Viber* is operated by the Japan-based multinational company Rakuten Inc. *Messenger* is a feature of Facebook’s main platform and offers instant messaging as well as audio and video calls between the platform’s users. *Zoom* is another US-based videotelephony service.

Participants were asked how 1. they, and 2. their clients, “experienced necessary urgent examinations, treatments, and therapies at a distance” (rated on a Likert scale 1 = negative, 2 = rather negative, 3 = rather positive, 4 = positive, 5 = I do not know).

Participants were asked about the possibility of reimbursement of the HCD services they provided by health insurance (yes, no, I do not know).

Regarding desired training opportunities when carrying out HCD, participants could choose among multiple answers in terms of knowledge about 1. infrastructure, 2. applications (apps), 3. law and data protection, 4. reimbursement by health insurance, 5. federal and cantonal ordinances, 6. client needs, 7. client requirements, 8. effectiveness, 9. communication methods, 10. the examination and treatment process, and 11. suitable methods. Similarly, participants could choose multiple answers with respect to the need for training opportunities within these 12 topics. Moreover, OTs and midwives reported with comments to open-ended questions what they perceived as advantages and disadvantages of HCD. Answers to questions that allowed for commentary on further desired support are not reported here.

The questionnaire was available in German, French, and Italian (the three languages spoken as a first national language by 63.5, 22.5 and 8.1% of inhabitants in Switzerland, respectively) [25]. All three language versions were translated and checked by a native speaker. The full questionnaires are provided in the annex. Skipping questions was not possible except for the last four questions concerning advantages and disadvantages of therapy at a distance and desired support/need for training opportunities.

The questionnaire was driven by the immediate need for survey data during the COVID-19-induced lockdown. As it is a new tool, psychometric properties regarding the construction, validity, and reliability of measurement could not be collected [26]. Interview questions were formulated jointly by two OTs and two midwives and were proofread, commented on, and completed by members of each professional association. One OT and one midwife not involved in the study filled out a test version and had only some minor suggestions for changes (e.g., regarding response options for area of work).

As the data was anonymized, Institutional Review Board approval was not required.

#### Statistical analysis

We used descriptive statistics including frequency distributions and means and standard deviations to analyze sociodemographic data. We calculated frequency distributions to describe the respondents’ use of media, their opinions of the media’s applicability, their experience of HCD, reimbursement of services, and desired training opportunities. We performed chi-square tests of independence to examine differences in media use patterns between OTs and midwives.

We tested data for Gauss-Markov theorems 1 to 6. To test for the first hypothesis (“profession, age in years, and area of work have an influence whether HCD was provided”) we performed a binary logistic regression analysis. To test the second hypothesis (“profession, age in years, area of work, reimbursement, and application used have an influence on experience of this HCD service”) we performed an ordinal logistic regression. We calculated response and cooperation rates according to Smith [27]. Missing data were deleted listwise. We performed statistical analysis with IBM SPSS Statistics 26®.

#### Analysis of qualitative data

The analysis of answers to the open questions was based on the principles of the Quantitative Content Analysis according to Früh [28]. The category scheme was derived from the data itself. Based on the repeated review of all answers to the question about the advantages and chances of HCD (*n* = 1052), six clearly distinguishable categories were formed, each containing different topics/motives. The assessment of all answers to the question about the disadvantages and limitations of HCD (*n* = 1129) resulted in five categories, one of which comprised three sub-categories. We concentrated the analysis on the first points mentioned in the answers, since this is the most spontaneous reaction to the question and most likely reflects what is seen as most important for the respondents.

## Results

The online questionnaire was completed by 1.269 health professionals, with OTs and midwives making up roughly half of respondents, respectively (OTs: *n* = 639; midwives: *n* = 630). Response rate (i.e., the number of complete interviews divided by the number of eligible contacts) was 22.5%, cooperation rate (i.e, the proportion of complete interviews of all responding contacts) was 52.6% [27].

### Sociodemographic information

Respondents had a mean age of 45.5 years and a mean professional experience of 19.5 years. The subsample of midwives was slightly older (46.2 years) and had longer professional experience on average (20.5 years) than the subsample of OTs (44.7 and 18.5 years, respectively). The respondents were predominantly female (96.6%), reflecting the gender distribution in these professions. 73% were German-speaking, 22.4% were French-speaking, and 4.6% were Italian-speaking, roughly reflecting the distribution of languages in the general Swiss population of 62.2% German, 22.9% French, and 8.0 Italian, with a slight overrepresentation of German-speakers, especially in the midwife subsample. More midwives than OTs worked in clients’ home settings (87.1 and 39.4%) and in inpatient care (37.0 and 25%). In contrast, more OTs worked in outpatient care (76.8 and 52.9%) and in school settings (19.2 and 1.4%) compared to midwives.

### Provision of health care at a distance

67.4% of all OTs (*n* = 431) and 79.5% percent of midwives (*n* = 501) provided HCD during the COVID-19 pandemic lockdown (total sample: 73.4%, *n* = 932). Seven respondents (six OTs, two midwives) who denied providing HCD nevertheless provided information on media they used for providing such services, bringing this subsample to a total of 940 respondents. Out of the forms of media that were used for HCD, the form that was used most was phone, followed by chat services, email, videotelephony, and SMS (see also Table 1).

**Table 1:** Media used for service provision at a distance

	Occupational Therapists (*n* = 437)	Midwives (*n* = 503)	Total sample (*n* = 940)	Chi-Square test		
				*X*^*2*^=	*df*=	*p*=
Use of phone *n(%)*	397 (90.8%)	491 (97.6%)	888 (94.5%)	21.37	2	< 0.001*
Use of chat services *n(%)*	201 (46.0%)	382 (76.1%)	583 (62.1%)	91.69	2	< 0.001*
Use of email *n(%)*	303 (69.3%)	219 (43.6%)	552 (55.6%)	64.31	2	< 0.001*
used videotelephony *n(%)*	226 (51.7%)	275 (54.8%)	501 (53.4%)	2.620	2	0.270
Use of SMS *n(%)*	158 (36.2%)	333 (66.3%)	491 (52.3%)	87.05	2	< 0.001*

The midwives subsample had one missing value for “use of phone” and two missing values for each other category. *X2* Chi-Square value, *df* degrees of freedom; * = *p*-value is significant at 0.05 level

#### Hypothesis 1: profession, age, and area of work have a significant and meaningful influence on whether HCD was provided

HCD was more likely to be provided by midwives compared to OTs (OR = 2.2; *p* < .001), and by those who worked in the home environment (OR = 1.6; *p* < .001) and in the outpatient area (OR = 2.4; *p* < .001). It was less likely to be provided by those who worked in inpatient settings (OR = .5; *p* < .05). “School setting” as area of work and age had no influence on whether HCD was provided or not. However, age correlated positively with use of SMS (standardized coefficient beta β_j_ = .16, *p* ≤ .001) and chat services (β_j_ = .09, *p* = .001), and negatively with the use of videotelephony (β_j_ = −.14, *p* < .001). Use of phone was independent from age.

Accordingly, the areas of knowledge where most respondents desired support when providing HCD were reimbursement by health insurance (70.8%) followed by law and data protection (60.4%). Roughly a third of respondents desired support regarding knowledge about suitable methods (34.4%), applications (32.5%), effectiveness (31.6%), and cantonal and federal ordinances (31.4%), respectively. More than half of respondents voiced a need for training opportunities in the following areas: reimbursement (65.6%), law and data protection (64.4%), and effectiveness (52.2%). OTs seemed to clearly voice a greater need for education about applications (51.5%) than midwives (31.4%). See also Table 2.

**Table 2:** Desire for training opportunities

	Occupational Therapists (*n* = 639)	Midwives (*n* = 630)	Total sample (*n* = 1269)
Education about reimbursement *n(%)*	430 (67.3%)	402 (63.8%)	832 (65.6%)
Education about law and data protection *n(%)*	425 (66.5%)	392 (62.2%)	817 (64.4%)
Education about effectiveness *n*(%)	368 (57.6%)	294 (46.7%)	662 (52.2%)
Education about suitable methods *n(%)*	338 (52.9%)	261 (41.4%)	599 (47.2%)
Education about applications *n(%)*	329 (51.5%)	198 (31.4%)	527 (41.5%)
Education about communication methods *n(%)*	256 (40.1%)	210 (33.3%)	466 (36.7%)
Education about cantonal and federal ordinances *n(%)*	207 (32.4%)	204 (32.4%)	411 (32.4%)
Education about client needs *n(%)*	171 (26.8%)	226 (35.9%)	397 (31.3%)
Education about examination/treatment process *n(%)*	188 (29.4%)	195 (31.0%)	383 (30.2%)
Education about client requirements *n(%)*	165 (25.8%)	142 (22.5%)	307 (24.2%)
Education about infrastructure *n(%)*	172 (26.9%)	124 (19.7%)	296 (23.3%)

Those who provided HCD were more likely to desire support regarding reimbursement by health insurance (OR = 1.6; *p* < .001) and regarding law and data protection (OR = 1.5; *p* < 0.001). Desire for knowledge about the remaining topics had no influence on whether HCD was provided or not.

#### Hypothesis 2: profession, age, area of work, possibility of reimbursement by health insurance, and application used have significant and meaningful influence on experience of this HCD service

The question on how healthcare was experienced at a distance during the COVID-19 pandemic lockdown was answered by 933 respondents. Among OTs, 67.5% experienced it as positive or mostly positive (*n* = 292), while 27.0% (*n* = 117) experienced it as negative or mostly negative. Among midwives, only 39.5% experienced it as positive or mostly positive (*n* = 198), while 57.5% (*n* = 288) experienced it as negative or mostly negative.

OTs experienced HCD significantly more positive than midwives (log odds = 1.3; *p* ≤ .001). Furthermore, videotelephony (log odds = 1.1; *p* ≤ .001) and use of phone (log odds = 0.8; *p* = .011) were positive predictors for positive experience of HCD, while use of SMS (log odds = − 0.33; *p* = .022) was a negative predictor. Age, area of work, use of email and chats had no significant influence on experience. However, there was a non-significant tendency (log odds = .43; *p* = .060) for reimbursement by health insurance as a positive predictor.

The question concerning their ability to get reimbursed for HCD was answered by 933 respondents. Of these, 17% (*n* = 159) stated that they were able to be reimbursed for these services and 55.4% (*n* = 517) stated that they were partially able to be reimbursed, and 15.2% (*n* = 142) stated that were not able to be reimbursed. Those numbers were similar for both professions.

**Fig. 1:**
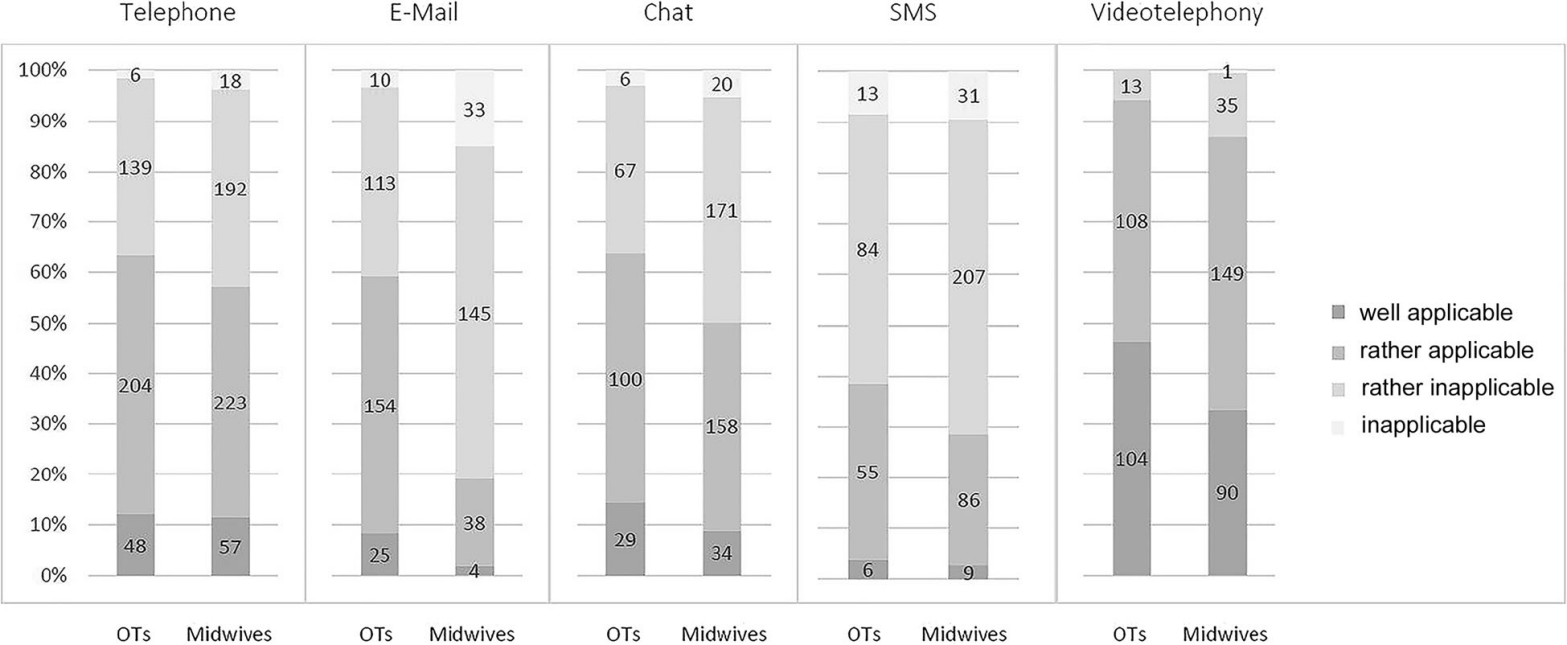
Applicability of different media for HCD. Numbers inside bars designate number of respondents

The applicability of media used for HCD was rated only by those respondents who had used the media in question. Applicability was rated on an ordinal 4-point scale (well applicable, rather applicable, rather inapplicable, or inapplicable). Videotelephony was the medium deemed applicable or well applicable by the highest percentage of respondents (90.2%), followed by phone (59.9%), and chat (54.9%). While email was deemed well or rather applicable for HCD by 59.3% of responding OTs, only 19.1% of responding midwives chose these answers (see Fig. 1). The respondents’ most used videotelephony apps were *WhatsApp* (67.5%), *Skype* (56.9%), *Zoom* (50.1%), and *Facetime* (41.1%), while only few used *MS Teams* (8.0%), *Messenger* (6.4%), or *Viber* (0.6%). *Doxy.me* was not used by any respondent.

### Perceived advantages and disadvantages

We received 1052 comments about advantages and 1162 about disadvantages in relation to HCD from 583 OTs and 578 midwives. The most frequently mentioned (39.3%) advantage of HCD is the possibility to maintain the relationship with clients and to carry out consultations under extraordinary circumstances such as the COVID-19 pandemic. Other advantages often named were the reduction in the (unpaid) workload through the simpler clarification of low-level questions from clients and through the elimination of the largely unpaid travel time (18.7%), as well as the fact that HCD gave certain clients easier access to health care (16.7%).

Several respondents stating advantages stressed that the benefits of HCD were limited and that HCD was no substitute for direct care. Some (12.7%) denied any advantages of HCD.

In the statements on the disadvantages, two themes predominated: First, OTs and midwives saw themselves as considerably restricted in their ability to recognize and assess the complexity of the situation as a whole due to the lack of physical presence (42.9%). Second, for OTs and midwives a large number of examinations, interventions, and therapies were hardly possible at a distance due to absence of direct physical interaction (31.9%). As a result, they would only have access to part of the available information, which could sometimes lead to situations that felt difficult for both sides. The importance of scent and touch for the intervention and care work was particularly emphasized: “Hands-on is an indispensable part of work”, OTs typically noted, whereas a midwife emblematically remarked: “Smelling, touching do not work via videotelephony”. The perceived limitations to relationship building, seen as an essential prerequisite for a successful intervention and care (10.1%), as well as the added value of presence for the instructional work (showing, guiding) were also repeatedly mentioned (6.7%). In addition, entire client groups were being disadvantaged from the perspective of the respondents (7.1%). They reported that this was especially true for clients with mental health and anxiety problems, clients who lack technical proficiency, clients without sufficient language skills, and/or who have a shy and inhibited demeanor.

## Discussion

This was the first study investigating HCD among OTs and midwives in the context of the COVID-19 pandemic in Switzerland. There is evidence that HCD is effective in assessing and improving clients’/patients’ health conditions [18]. However, to date, clinical studies have largely been conducted with complex telemedicine systems or stand-alone software programs. These were often explained to the users beforehand, i.e., to health care providers and clients/patients in order to familiarize them with the system, the system’s equipment, and the treatment procedures [18]. A lack of training might influence users’ adoption and/or increase their resistance to HCD. The COVID-19 pandemic lockdown required health professionals to provide their services remotely without prior training or education.

Our first hypothesis that profession, age, and area of work had a significant and meaningful influence on whether HCD was provided, was partially proven. About two out of three OTs and four out of five midwives provided services remotely. Not surprisingly, HCD was used mainly for care in the outpatient and home environments. Our assumption that age has an influence on provision, as technological affinity may decrease with age, was not proven. However, the younger the caregiver, the more likely videotelephony was used. In contrast, older caregivers preferred SMS and chat services when providing services and care.

It has been described in the literature that the implementation of novel telemedicine solutions can pose difficulties [29]. Chat services seem to be the most frequently used novel communication technology and deemed most applicable out of all these services, possibly due to their interactiveness. Furthermore, they are common within private use, meaning users are more likely to be familiar with them, thus lowering the threshold when applying them in professional settings. However, chat services were not reimbursed by health insurances.

With the phone being the most ubiquitous and low threshold communication technology available, it is no surprise that it was the medium most frequently used for HCD by health professionals during the COVID-19 pandemic lockdown. However, videotelephony was seen as being much more applicable. This may be the case as videotelephony more closely resembles in-person encounters contrasted to audiotelephony alone. Despite its apparent applicability, videotelephony was only used by about half of respondents who performed HCD. This may be due to a lack of familiarity with the relevant technology and/or data protection concerns on the part of the health professionals and/or their clients. In fact, law and data protection seem to be a concern among health professionals. Education and advocacy are described as central to the continued use of HCD [8]. Legal issues (concerning laws, regulations, guidelines, and standards) regarding confidentiality, operational or contractual requirements, billing and coding processes, clinical and nonclinical documentation, licensure regulations, clients rights and responsibilities, and data protection and secure data storage are all concerns that must be addressed before implementing HCD [8].

Although OTs provided fewer HCD services than midwives, their experience of HCD was more positive. Interestingly, whether the remote service was reimbursed by the health insurance provider only played a minor role (non-statistically significant tendency) for the experience of HCD. The Swiss Federal Office for Public Health (FOPH) launched a factsheet on reimbursement of costs for HCD during the COVID-19 pandemic on April 6, 2020 which was valid until June 22, 2020 [30]. The factsheet stated that a brief midwife consultation by phone could only be invoiced if services were provided within the scope of the FDHA Ordinance on Benefits in the Compulsory Health Insurance Scheme. During the lockdown, remote services replaced attendance services. Reimbursement was restricted to five short consultations by phone per client. Midwives’ services that could be provided at a distance were limited to comprehensive advice during pregnancy, pregnancy-related complaints, care in the postpartum period, and breastfeeding advice. For OTs, HCD were reimbursed only following prior initial face to face consultation or treatment. Moreover, HCD could be provided if clients showed symptoms of a respiratory tract infection, belonged to the group of particularly endangered persons, or travel/transport could not be guaranteed while adhering to the necessary hygiene measures. Services had to be limited to what the client could do independently or with the support of a caregiver without the use of unavailable aids and without physical interaction (such as touch for assessment) with the therapist.

HCD reimbursement appeared to be a major concern for health care professionals, as reflected in the open-response section of the survey and in the preferences selected regarding support and continued teaching. HCD were only reimbursed if they were carried out via videotelephony (a phone consultation alone was not reimbursed) and a maximum of 30 min a day were deemed billable. This restriction is not reflected in a greater use of videotelephony by OTs, but it may be reflected in their greater desire for education about applications.

Accordingly, the most desired topic for training opportunities in relation to HCD was education about reimbursement. About three quarters of respondents who performed HCD stated they could not be or could only partially be reimbursed for these services by insurers. This was the case for both OTs and midwives alike.

The convenience aspect of HCD, mainly concerning the elimination of travel and waiting times, and the ability to carry out consultations from the comfort of one’s home at any time have been described in many earlier studies [18].

A considerable number of the responding OTs and midwives had concerns about the use of HCD. They associated HCD with restricted professional abilities and lowered effectiveness. This is in line with findings of other studies about health care professionals’ perceptions of HCD  [31–33]. However, since the statements are founded on specific experiences with HCD during the COVID-19 pandemic, results should be interpreted with caution and cannot be generally related to HCD. In our study, OTs and midwives had not systematically received prior introduction and training to available systems nor technical or emotional support. Furthermore, during the pandemic HCD did not complement traditional care but often had to replace it, meaning that the primary aim was not only consultation but also therapy/care. Accordingly, most negative comments criticised the lack of physical interaction with HCD. The apparent discrepancy of rated experience (i.e., about half of respondents rated the experience of HCD as positive or rather positive) and comments (i.e., predominantly negative) may reflect both the appreciation of HCD as a temporary solution during the extraordinary pandemic situation and concerns about efforts to replace traditional care in the long-term by HCD.

Of particular interest was the scepticism noted in the comments. This conflicts with the attitude of OT and midwifery students who associate digital media primarily with the promise of efficiency [34].

The questionnaire focused on the attitude of the health care professionals. As a next step, it would be important to ask patients and clients about their experiences with HCD during the pandemic to optimize HCD provisions.

The data provided is only a snapshot of how the situation was perceived by OTs and midwives at a certain time (the lockdown began in Switzerland on March 16, 2020; the survey was accessible from May 11, 2020 to May 26, 2020). Furthermore, the generalizability of the results is limited, as only about one in five of the contacted OTs and midwives filled out the survey completely within the short open time window of 16 days. We also cannot exclude the existence of bias, e.g., the probability that health professionals with a distinct opinion (either positive or negative) or those who had (or had not) provided HCD were more likely to answer the survey. Nevertheless, with more than 1200 participants, the results provide valuable insights into the perceived experiences and challenges of HCD as a result of the COVID-19 pandemic.

The application of information and communication technologies (ICT) for remote health care is nothing new, with some initial efforts already dating back to the 1980’s [35]. Several national health sectors have been adopting technological innovations in telemedicine for years [36–38]. The COVID-19 lockdown triggered a wide use of HCD, and it is to be expected that application of HCD will grow beyond the time frame of the current pandemic. As the two professions (OT and midwifery) require physical interaction and touch for assessment and therapy of their clients, HCD will likely serve as a tool to complement conventional therapy and not act as a substitute. Going forward, we suggest offering workshops for OTs and midwives in which the representative barriers and facilitators and the desired support respective to HCD are addressed.

## Supplementary Information

**Additional file 1:.** Study Questionnaire. Survey on opportunities and limits regarding health care at a distance during the Covid-19-pandemic.

## Data Availability

The dataset supporting the conclusions of this article is available upon request from the corresponding author.

## Abbreviations

COVID-19: Coronavirus disease 2019
HCD: Health care at a distance
OT: Occupational therapist
