# Supplementary material for: Does therapy always need touch? A cross-sectional study among Switzerland-based occupational therapists and midwives regarding their experience with health care at a distance during the COVID-19 pandemic in spring 2020
Source: BMC Health Serv Res. 2021 Jun 15;21:578. doi: 10.1186/s12913-021-06527-9 (PMC8205206; doi:10.1186/s12913-021-06527-9)
Supplement: Supplementary file 1 — Additional file 1:. Study Questionnaire. Survey on opportunities and limits regarding health care at a distance during the Covid-19-pandemic. [file 12913_2021_6527_MOESM1_ESM.pdf]

BHSR-D-20-02562
Does Therapy Always Need Touch? - A cross-sectional study among Switzerland-based occupational therapists and midwives regarding their experience with health care at a distance during the COVID-19 pandemic in Spring 2020 Verena Klamroth, M.D.; Michael Gemperle, PhD; Thomas Ballmer; Susanne Grylka-Baeschlin, PhD; Jessica Pehlke-Milde; Brigitte Gantschnig

Study Questionnaire

Survey on opportunities and limits regarding health care at a distance during the Covid-19-pandemic.

Instructions for Questionnaire. Please read carefully before you begin.

The Covid-19 pandemic has turned the digital future into the present. In a short amount of time, in-person examinations, treatments and therapies had to be replaced by health care at a distance. Health professionals did not only have to treat clients effectively, but also had to adhere to the relevant laws and regulations. We would like to ask you a few questions regarding your experiences with health care at a distance during the Covid-19 pandemic. Based on your answers, we would like to develop needs-based recommendations, in cooperation with the professional associations.

What is this questionnaire about?

The aim of this survey is to examine your experiences during the Covid-19 pandemic and your needs regarding examinations, treatments and therapies at a distance.

How long does it take to answer the questions?

Answering the questions takes approximately 10 minutes.

How should you fill out this questionnaire?

- Start with the first question, then continue in the order the questions are asked.
- Tick the box corresponding to your answer. In some questions, multiple answers are possible.
- Some questions require a numerical answer, some allow for you to write a short text.
- There are no wrong answers.

I have read the information regarding this questionnaire and agree to take part:

Yes

No

1. Personal data

Age in years:

Professional experience in years:

Profession: ☐Occupational Therapist ☐Midwife

My institution/organisation is mainly active in the following sector:

☐ Outpatient health care (e.g. private practice)

☐ Inpatient health care (e.g. clinic, care home)

☐ Home health care

☐ School-based health care

2. During the Covid-19 pandemic, did you perform necessary, urgent examinations, treatments and therapies at a distance instead of in person?
   Yes
   No

3. Which media did you use to perform necessary, urgent examinations, treatments and therapies at a distance (multiple answers possible)?

   Telephone:

   ☐ Yes

   ☐ No

   In your opinion, how applicable is the telephone for performing necessary, urgent examinations, treatments and therapies at a distance?

   ☐ inapplicable

   ☐ rather inapplicable

   ☐ rather applicable

   ☐ well applicable

   E-mail

   ☐ Yes

   ☐ No

   In your opinion, how applicable is e-mail for performing necessary, urgent examinations, treatments and therapies at a distance?

   ☐ inapplicable

   ☐ rather inapplicable

   ☐ rather applicable

☐ well applicable

Chat (e.g. WhatsApp)

☐ Yes

☐ No

In your opinion, how applicable are chat-services for performing necessary, urgent examinations, treatments and therapies at a distance?

☐ inapplicable

☐ rather inapplicable

☐ rather applicable

☐ well applicable

Short Message Services (e.g. SMS)
In your opinion, how applicable are short message services for performing necessary, urgent examinations, treatments and therapies at a distance?

☐ inapplicable

☐ rather inapplicable

☐ rather applicable

☐ well applicable

Videotelephony
In your opinion, how applicable are videotelephony services for performing necessary, urgent examinations, treatments and therapies at a distance?

☐ inapplicable

☐ rather inapplicable

☐ rather applicable

☐ well applicable

4. Which digital applications (apps) for videoteleohony have you used in ordert o perform necessary, urgent examinations, treatments and therapies at a distance?

Doxy.me

☐ Yes

☐ No

Skype

☐ Yes
☐ No

Viber

☐ Yes
☐ No

WhatsApp

☐ Yes
☐ No

MS Teams

☐ Yes
☐ No

Facetime

☐ Yes
☐ No

Messenger

☐ Yes
☐ No

Zoom

☐ Yes
☐ No

Others

☐ Yes
☐ No

5. How did you experience necessary, urgent examinations, treatments and therapies at a distance?
   ☐ negatively
   ☐ mostly negatively
   ☐ mostly positively
   ☐ positively
   ☐ I don’t know

6. In your opinion, how did your clients expeirence necessary, urgent examinations, treatments and therapies at a distance?
   ☐ negatively
   ☐ mostly negatively
   ☐ mostly positively
   ☐ positively
   ☐ I don’t know

7. Were you able to be reimbursed for necessary, urgent examinations, treatments and therapies at a distance?
   ☐ Yes
   ☐ No
   ☐ Partially

☐ I don't know

8. In your opinion, what are the opportunities and advantages of examinations, treatments and therapies at a distance?

   Please note here (max. 100 characters):

9. In your opinion, what are the limits and disadvantages of examinations, treatments and therapies at a distance?

   Please note here (max. 100 characters):

10. What support do you/would you have wished for when performing necessary, urgent examinations, treatments and therapies at a distance? (multiple answers possible):

    ☐ Knowledge about infrastructure
    ☐ Knowledge about applications (apps)
    ☐ Knowledge about law and data protection
    ☐ Knowledge about reimbursement
    ☐ Knowledge about cantonal and federal ordinances
    ☐ Knowledge about client needs
    ☐ Knowledge about client requirements
    ☐ Knowledge about effectiveness
    ☐ Knowledge about communication methods
    ☐ Knowledge about examination/treatment process
    ☐ Knowledge about suitable methods
    ☐ Knowledge about other topics (please name)

11. If there were training opportunities regarding necessary, urgent examinations, treatments and therapies at a distance, in your opinion, what topics would be important (multiple answers possible):

    ☐ Education about infrastructure
    ☐ Education about applications (apps)
    ☐ Education about law and data protection
    ☐ Education about reimbursement
    ☐ Education about cantonal and federal ordinances
    ☐ Education about client needs
    ☐ Education about client requirements
    ☐ Education about effectiveness
    ☐ Education about communication methods
    ☐ Education about examination/treatment process
    ☐ Education about suitable methods
    ☐ Education about other topics (please name)

12. This was the last question. Do you have any other comments on the topic of necessary, urgent examinations, treatments and therapies at a distance?

    Please note here (max. 100 characters):
